# Ammonia Diffusion through Nalophan Double Bags: Effect of Concentration Gradient Reduction

**DOI:** 10.1155/2014/214190

**Published:** 2014-11-24

**Authors:** Selena Sironi, Lidia Eusebio, Laura Capelli, Emanuela Boiardi, Renato Del Rosso

**Affiliations:** Department of Chemistry, Materials and Chemical Engineering “Giulio Natta”, Politecnico di Milano, 20133 Milan, Italy

## Abstract

The ammonia loss through Nalophan bags has been studied. The losses observed for storage conditions and times as allowed by the reference standard for dynamic olfactometry (EN 13725:2003) indicate that odour concentration values due to the presence of small molecules may be significantly underestimated if samples are not analysed immediately after sampling. A diffusion model was used in order to study diffusion through the bag. The study discusses the effect of concentration gradient (Δ*C*) across the polymeric membrane of the analyte. The Δ*C* was controlled by using a setup bag called “double bags.” Experimental data show a reduction of ammonia percentage losses due to the effect of the external multibarrier. The expedient of the double bag loaded with the same gas mixture allows a reduced diffusion of ammonia into the inner bag. Comparing the inner bag losses with those of the single bag stored in the same conditions (*T*, *P*, *u*) and with equal geometrical characteristics (*S*/*V*, *z*), it was observed that the inner bag of the double bag displays a 16% loss while the single bag displays a 37% loss. Acting on the Δ*C* it is possible to achieve a gross reduction of 57% in the ammonia leakage due to diffusion.

## 1. Introduction

The development of both industrial and agricultural areas, coupled with the increase of population density, has brought about in some regions the forced coexistence between production sites and residential areas. One of the outcomes of this fact is the increased attention on air quality, involving detailed monitoring of ambient air in the area. Specifically, the focus is on the olfactory nuisance, which considered as a consequence of the chemical plants operation urged the scientific community and the lawmakers to consider odour as an air pollutant. In fact it has been found that the olfactory nuisance, besides being annoying for the population, may be the direct cause of several pathologies such as nausea, headache, migraine, and psychological discomfort for the citizens living in the surroundings of an industrial or agricultural productive site.

Due to the necessity of preserving an acceptable air quality level, especially in residential areas close to industrial plants, that are odour-emitting sources, a scientific methodology was devised in order to reliably quantify odour, the so-called dynamic olfactometry [1]. Thus the European Community stepped in producing a regulation concerning this new science, the norm EN13925 [2].

With olfactometric analysis it is possible to quantify odour concentration in air samples coming from the sites of interest by means of a panel of people chosen in accordance with the norm EN1375 [2]. Due to the complications entailed with on-site olfactometric analyses, samples are usually collected at the source and then stored in a suitable container until they are analysed in a dedicated laboratory [1, 3–6].

As for the recovery and conservation of the odorous compounds the standard prescribes to use bags with specific characteristics in order to avoid as much as possible contamination of sample, deterioration, and osmotic diffusion out of the bags [7].

The sample bags must meet the quality requirements of EN 13725, in order to preserve the odour sample for (at least) 30 hours.

The European Standard on dynamic olfactometry (EN13925) [2] states the general requirements relevant to the materials used for the realization of sampling equipment. According to the European Standard, the materials used for storing compounds for olfactometric analyses shall be odourless; furthermore, they shall be selected so to minimize the physicochemical interactions between sample components and bag materials, also the materials ought to have minimal permeability in order to reduce sample losses caused by diffusion, and last but not least the bag should have a smooth internal surface.

The materials allowed for the manufacturing of sample containers (bags), as listed in Section 6.3.1 of the actual standard, are tetrafluoroethylene hexafluoropropylene copolymer (FEP), polyvinylfuoride (PVF, Tedlar), and polyethyleneterephthalate (PET, Nalophan).

According to the European Standard these materials should be tested for suitability, by verifying whether or not they can store a mixture of odourants with minimal variations in odour and composition for times of storage of 30 hours, which is the maximum storage time allowed by the European Standard.

Lately a lot of researches have been done on these “allowed bag materials” prescribed by the norm [8–22] in order to evaluate their performances. Results highlighted a nonnegligible diffusion across the bag wall with respect to certain substances, particularly for small or water-soluble molecules such as ammonia (NH_3_) and hydrogen sulphide (H_2_S) [7, 10, 11, 14, 23, 24].

For these reasons, the new German guideline for odour sampling (VDI 3880) limits sample storage to 6 h only, as also discussed by Laor et al. [25, 26].

Apart from polymeric films like Tedlar, Nalophan, and Teflon, many studies [5, 8–10, 14, 17, 18, 21, 22] focused on researching both new materials and new superficial treatments to apply to the films, already contemplated in the norm EN13725, in order to obtain a suitable product for sampling and storage of odorous gaseous mixtures, usually made of volatile organic compounds (VOC), capable of meeting the strict requirements imposed by the European regulation.

Kim et al. [5, 8] have performed an assessment of poly-coupled films polyester-aluminium (PEA) performances. Comparing the results obtained with PEA and those obtained with Tedlar, it was highlighted that the first one assures higher recovery as far as nonaromatic compounds are concerned [8]. Other researchers have studied the behaviour of polymeric films such as PET, both with a plasma [8] superficial treatment [21, 22] and with a poly-coupling with different materials (e.g., FlexFoil (PET-NY-AL-CPE) [9, 10, 14, 18]). Anyway, to this day a material has not yet been found suitable for effectively storing for 30 hours a gaseous mixture of VOCs like those that can be encountered in many different problematic industrial areas such as landfills and dumps, foundries, intensive rearing sites, biomass anaerobic digestion plants, and refineries. The diffusion of a gaseous mixture through polymeric films is driven by a large number of different factors; here the most significant ones are reported as an example:

- the characteristics of the polymeric film constituting the storage bag, both geometrical and physicochemical, such as the wall thickness, the crystallinity grade of the polymer, and the orientation of the polymeric structure [7, 27–29];
- the bag's surface to filling volume ratio (Fick's law) [24];
- the conditions at which the sample is stored (temperature and humidity) and the concentration gradient across the membrane, that is, the film (Permeability law, Fick's law) [23].

The aim of the present work is to investigate a new possible bag design in order to minimize the diffusion of small water-soluble molecules through Nalophan films. The original idea is a new structure bag design called “double bag” that is particular sampling bag made of two concentric films barrier filled with the same gaseous mixture. For this purpose, even high concentration of ammonia does not play a fundamental role in the total odour measured, and the behaviour of a small molecule with a structure similar to the water as ammonia is considered. The final goal is, starting from the analysis of Fick's law, to mitigate diffusive phenomena trying to reduce as much as possible the difference in concentration (Δ*C*) across the polymeric membrane of the analyte.

## 2. Materials and Methods

### 2.1. Materials

The Nalophan polymer used to realize the bags employed for the experimental tests consists of a one-layer foil of polyterephthalic ester copolymer with 20 *μ*m thickness supplied by Tilmmanns S.p.A (Milan, Italy).

**Figure 1:**
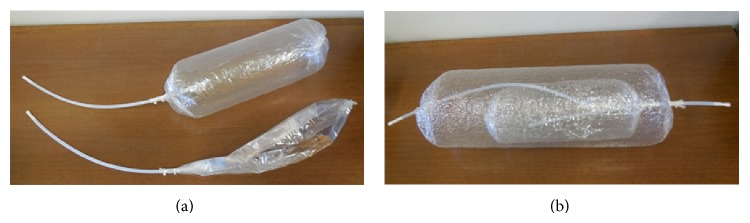
Nalophan bags: (a) single bag and (b) double bags.

A single bag was obtained starting from a tubular film with a diameter of 22.5 cm, which was then cut in different lengths. One end was equipped with a clamp closure while the other end was provided with a Teflon inlet tube for sample collection (Figure 1(a)).

The double bags were obtained starting from two different tubular films cut in different lengths. The inner bag was obtained from a tubular film with a diameter of 22.5 cm, while the outer one was obtained from a tubular film with a diameter of 31 cm.

The double bag is a two-concentric-bags design composed (Figure 1(b)) of an inner bag with a capacity of 6000 cm^3^ and surface equal to 2580 cm^2^ and an outer bag with a capacity of 12000 cm^3^ and surface equal to 5208 cm^2^. The different volumes are meant to create an interspace between the two bags. The double bag was realized following this procedure,

- One end of the inner bag was equipped with a clamp closure while the other end was provided with a Teflon inlet tube for sample collection.
- The outer bag was clamped on the Teflon inlet tube of the inner bag and on the other side was provided with a Teflon inlet tube for sample collection.

The NH_3_ decay over time was evaluated using a gas chromatography (GC) technique for the quantification of NH_3_ concentration inside the bag. The ammonia concentration was measured using a HP Agilent 6890 gas chromatograph equipped with an Agilent HP-5MS fused silica capillary column (CP 7591-PoraPlot Amines, length 25 m, internal diameter 0.32 mm, and film thickness 10 *μ*m). The oven temperature follows a three-step program: 100°C for 12 minutes, from 100°C to 200°C with a rate of 8°C/min, and 200°C for 5 minutes. The carrier gas was helium with a constant flow of 3 mL/min (a pressure of 1.21 atm and a mean velocity of 53 cm/s). The gaseous mixture inside the bags was analysed with the GC, equipped with a TCD detector, at specific time intervals, in order to evaluate the variations of NH_3_ concentration (ppm) over time.

A calibration curve was traced to correlate the area of the GC peak with the NH_3_ concentration (ppm). Instrument calibration was performed analysing different standard concentrations of NH_3_ in air ranging from 10,000 to 60,000 ppm. Standards were obtained starting from different liquid mixtures of NH_3_ in water and analysing the headspace obtained in a fixed volume of air where the liquid was fluxed and then stored at a controlled temperature. The liquid phases were prepared at room temperature (20°C) mixing from 4 mL to 11 mL of a liquid solution of NH_3_ at a concentration of 30% w/w and 50 mL of distilled water according to Field and Combs [30].

All the tested samples were realized by filling the Nalophan bags with a gaseous mixture of ammonia in wet-air, with an ammonia concentration of about 55,000 ppm_V_ and a relative humidity of 60%, which will be defined as the “test mixture.” The high concentrations were chosen to stress ammonia diffusion phenomena through the film. The test mixture was prepared using the headspace technique. The liquid phase was prepared at room temperature (20°C) mixing 10.5 mL of a liquid solution of NH_3_ at a concentration of 30% w/w and 50 mL of distilled water.

During storage time, physical parameters like temperature and relative humidity were kept under control using a climatic chamber (Chamber GHUMY by Fratelli Galli, Milano, Italy).

### 2.2. Methods

All tests were performed measuring the NH_3_ concentration at different times after sample preparation. More specifically, NH_3_ was analysed, every hour, from 0 to 26 h. Each measurement involved the withdrawal of 300 *μ*L of the test mixture by means of a syringe and the injection of the taking in the GC.

The diffusion of ammonia was evaluated through Nalophan bags having, respectively,

- a capacity of about 6000 cm^3^ and a surface equal to 2580 cm^2^ for the single bag,
- a capacity of about 6000 cm^3^ and a surface equal to 2580 cm^2^ for the inner bag,
- a capacity of about 12000 cm^3^ and a surface equal to 5208 cm^2^ for the outer bag.

All the bags were filled with 6000 cm^3^ of the above defined test mixture and then stored at a constant temperature of 23°C and an external relative humidity of 60%. The external relative humidity was set equal to the internal relative humidity in order to avoid water diffusion during storage and its potential influence on ammonia diffusion.

## 3. Calculations

The diffusion phenomena through a polymeric film are described by Fick's law. According to this theory, the specific molar flow is defined as

(1) $$\begin{array}{c} j=-D\frac{\partial C}{\partial x}, \end{array}$$

where

- *j* is the specific molar flux (mol/m^2^/s),
- *D* is the diffusion coefficient (molecular diffusivity) of the compound through the film (m^2^/s),
- *C* is the concentration of the diffusing compound (mol/m^3^),
- ∂*x* is the differential thickness of the film.

The film thickness can be therefore expressed as

(2) $$\begin{array}{c} \overset{z}{\underset{0}{\int }}dx=z, \end{array}$$

where *z* is the film thickness (m).

In the expression above (1), only the main direction is considered, moving from the gradient to the single derivative of *C*.

**Figure 2:**
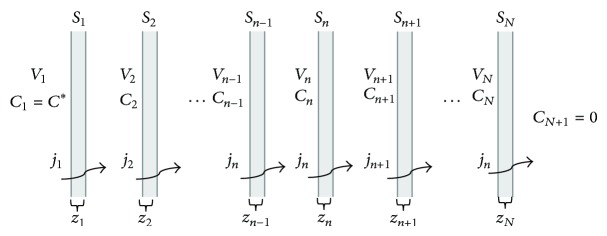
Schematization of diffusion through the multilayer thin film of the bag.

Referring to Figure 2, which schematizes the diffusion phenomenon through a multilayer thin film which constitutes the sampling bag, we can define the following:

- *S*_*n*_ is the surface of the *n*-polymeric film (m^2^);
- *z*_*n*_ is the thickness of the *n*-film (m);
- *C*_*n*_ is the concentration inside the *n*-volume (mol/m^3^);
- *C*_*N*+1_ is the concentration outside the film (mol/m^3^); for a single bag it is generally considered negligible (*C*_*N*+1_ = 0);
- *j*_*n*_ is the specific molar flow through the *n*-film (mol/m^2^/s).

If the film thickness can be considered negligible, then the accumulation term inside the material is negligible as well.

With this assumption, a generic *j*_*n*_ turns out to be constant across the film (*x* for 0 < *x* < *z*).

By integrating (1) in *dx* between 0 and *z*_*n*_, the specific molar flow *j*_*n*_ can be expressed as

(3) $$\begin{array}{c} j_{n}=-D\frac{C_{n+1}-C_{n}}{z_{n}}. \end{array}$$

Note that *j*_*n*_ is relevant to an infinitesimal portion of the exchange surface *dS*.

Assuming that the internal molar concentration of the *n*-bag, *C*_*n*_, is constant inside the whole internal volume *V*_*n*_ and also the external concentration *C*_*n*+1_ is constant inside the external volume *V*_*n*+1_, then the global flow *J* through the exchange surface *S*_*n*_ can be calculated by integrating as follows:

(4) $$\begin{array}{c} J=\overset{S_{n}}{\underset{0}{\int }}j_{n}\;dS \end{array}$$

(5) $$\begin{array}{c} J=S_{n}j_{n}. \end{array}$$

In order to obtain a general equation describing the diffusion trough the multilayer barrier system, a molar mass balance was written on the *n*-volume:

(6) $$\begin{array}{c} \frac{\partial M_{n}}{\partial t}=J_{n-1}-J_{n}. \end{array}$$

Combining (5) with (6), the evolution of molar content in the volume *V*_*n*_ may be calculated from the molar flow through the *n* surface and *n* − 1 surface:

(7) ∂Mn∂t=∂CnVn∂t=jn−1Sn−1−jnSn=−DSn−1Cn−Cn−1z+DSnCn+1−Cnz

(8) $$\begin{array}{c} \frac{\partial M_{n}}{\partial t}=-\frac{D}{z}\left({\left(S_{n-1}+S_{n}\right)C}_{n}-S_{n-1}C_{n-1}-{S_{n}C}_{n+1}\right). \end{array}$$

The general conditions applying both to the multilayer system and to the specific case, linking the two situations, are as follows:

- when *n* ≤ 1, it is considered that the concentration of the multilayer inner bag is equal to *C* ^*^,
- when 1 < *n* < *N*, it is considered that the concentration of the concentric bag containing the inner one is equal to *C*_*n*_,
- when *n* > *N*, it is considered that the concentration outside the outer bag of the multilayer system is negligible (*C*_*n*_ = 0),

where *N* is the number of the concentric bags.

The multilayer film model could represent the double bag considering a multilayer system with *N* equal to 2 (*N* = 2).

In the case considered of the double bag, the outer surface (*S*_*n*_) is twice the inner surface:

(9) $$\begin{array}{c} S_{n}=2S_{n-1}. \end{array}$$

Combining (8) and (9), considering the general conditions reported above, the molar flow through the inner bag (*n* = 1) can be expressed as

(10) ∂Mn∂t=∂CnVn∂t=−Sn−1Dz3C1−C1−2C2=−Sn−1Dz2C1−2C2

while the flow through the outer bag (*n* = 2) can be expressed as

(11) ∂Mn∂t=∂CnVn∂t=−Sn−1Dz3C2−C1−0=−Sn−1Dz3C2−C1.

Considering the same filling volume for the inner and the outer bags, the concentration trends over time will be described by the following expressions:

(12a) $$\begin{array}{c} \text{Inner}\;\text{Bag}:\frac{\partial C_{N-1}}{\partial t}=-\frac{S_{n-1}D}{zV_{N}}\left(2C_{N-1}-2C_{N}\right) \end{array}$$

(12b) $$\begin{array}{c} \text{Outer}\;\text{Bag}:\frac{\partial C_{N}}{\partial t}=-\frac{S_{n-1}D}{zV_{N}}\left(3C_{N}-C_{N-1}\right). \end{array}$$

Equations (12a) and (12b) constitute a system of differential homogeneous equations of the first order (ODE) that could be rewritten as

(13) $$\begin{array}{c} \text{Inner}\;\text{Bag}:y_{1}^{\prime }=2ky_{1}-2ky_{2},\\ \text{Outer}\;\text{Bag}:y_{2}^{\prime }=-ky_{1}+3ky_{2}, \end{array}$$

where

(14) $$\begin{array}{c} y_{i}^{\prime }=\frac{\partial C_{i}}{\partial t}\;\;k=-\frac{S_{n-1}D}{zV_{N}}\;\;y_{i}=C_{i}. \end{array}$$

The solution of the system of differential homogeneous equation of the first order is an exponential function such as

(15) $$\begin{array}{c} \vec{y}=\overset{n}{\underset{i=1}{\sum }}C_{i}e^{\lambda_{i}t}\vec{u_{k}}. \end{array}$$

The matrix's eigenvalues (*λ*_*i*_) are

(16) $$\begin{array}{c} \lambda_{1}=4k\;\;\lambda_{2}=k \end{array}$$

and the eigenvectors ($\vec{u_{k}}$) are

(17) u→1=−11  u→2=21.

The boundary conditions required for solving the equations system (13) can be set as

(18) $$\begin{array}{c} y_{1}\left(t\right)=C_{0}\;\text{per}\;\;t=0.\\ y_{2}\left(t\right)=C_{0}\;\text{per}\;\;t=0. \end{array}$$

Using *λ*_*i*_, $\vec{u_{k}}$ and the boundary conditions, substituting in (15), the concentration trends over time for the double bag follow these expressions:

(19) $$\begin{array}{c} C_{1}\left(t\right)=-\frac{1}{3}C_{0}e^{\lambda_{1}t}+\frac{4}{3}C_{0}e^{\lambda_{2}t},\\ C_{2}\left(t\right)=\frac{1}{3}C_{0}e^{\lambda_{1}t}+\frac{2}{3}C_{0}e^{\lambda_{2}t}, \end{array}$$

where

- *C*_1_(*t*) is the concentration trend for the inner bag,
- *C*_2_(*t*) is the concentration trend for the outer bag.

Moreover, for the double and single bag comparison purpose the multilayer film model could represent the single bag considering a multilayer system with *N* set equal to one (*N* = 1). In the case of a single bag, the surface *S*_*n*_ is equal to the surface *S*_*n*−1_. Combining (8) with the general condition reported above, the molar flow through the single bag (*n* = 1) can be expressed as

(20) $$\begin{array}{c} \frac{\partial M_{n}}{\partial t}=-\frac{DS_{n}}{z}C_{1}. \end{array}$$

The concentration trends over time can be then expressed as

(21) $$\begin{array}{c} \frac{\partial C_{1}}{\partial t}=-\frac{DS_{n}}{V_{n}z}C_{1}. \end{array}$$

The theoretical dissertation concerning the single bag is here simply reported, since it has been discussed in detail in a previous work [24].

The concentration trend over time for the single bag is

(22) $$\begin{array}{c} \frac{C}{C_{0}}=e^{-\left(S_{n}D/V_{n}z_{n}\right)t}. \end{array}$$

## 4. Results and Discussion

Table 1 shows ammonia concentration values measured in terms of ppms, at different instants in the time domain and ammonia loss in terms of percentage points for two different setups: a double bag (DB) and a single bag (SB).

Columns one and two in Table 1 contain the concentrations and the percentage losses of ammonia over time, respectively, for the inner bag (DB_in_ 6000–2580) with a *V*_*n*_ equal to 6000 cm^3^ and a *S*_*n*−1_ equal to 2580 cm^2^. Columns three and four show the concentrations and the percentage losses of ammonia over time, respectively, for the outer bag (DB_out_ 6000–5280) filled with a *V*_*n*_ equal to 6000 cm^3^ and a *S*_*n*_ equal to 5280 cm^2^. Columns five and six provide the concentrations and the percentage losses of ammonia over time, respectively, for the single bag (SB 6000–2580) with a *V*_*n*_ equal to 6000 cm^3^ and a *S*_*n*_ equal to 2580 cm^2^ just like the inner one for the double bag case.

Exploiting the system of (19), the percentage ammonia loss over time for the inner bag (NH_3loss%_inner_) and for the outer one (NH_3loss%_outer_) can be estimated being equal to, respectively,

(23) NH3loss%_inner=1−C1C0∗100=1+13eλ1t−43eλ2t∗100NH3loss%_outer=1−C2C0∗100=1−13eλ1t+23eλ2t∗100

while from (22) it is possible to compute the percentage loss of ammonia over time for the single bag:

(24) $$\begin{array}{c} {\text{NH}}_{3\text{loss}\%}=1-\frac{C}{C_{0}}{=1-e}^{-\left(S_{n}D/V_{n}z_{n}\right)t}. \end{array}$$

The analyses of these data provide some useful insights. The single bag scenario shows a loss of ammonia after 26 hours equal to the 37% of the initial amount. The percentage ammonia loss after 26 hours with respect to the inner bag is 16%. Since the inner bag is identical in size and filling to the single bag, the ratios (*S*/*V*) in the two cases will be also identical and equal to 0.43 cm^−1^. A comparison of the ammonia losses over time in the two situations highlights a significant difference: the ammonia loss is much smaller for the inner bag (16%) with respect to the one observed for the single bag (37%). Thus it is possible to say that reducing the transmembrane concentration difference (Δ*C*), across the polymeric film, achieved by making use of the double bag, the ammonia leakage can be effectively reduced, after 26 hours, by 57%.

Finally, the ammonia loss of the outer bag is 52%. Differences in ammonia loss for single bag (37%) and outer bag (52%) are due to the difference in *S*/*V* as observed by Sironi et al. [24].

**Figure 3:**
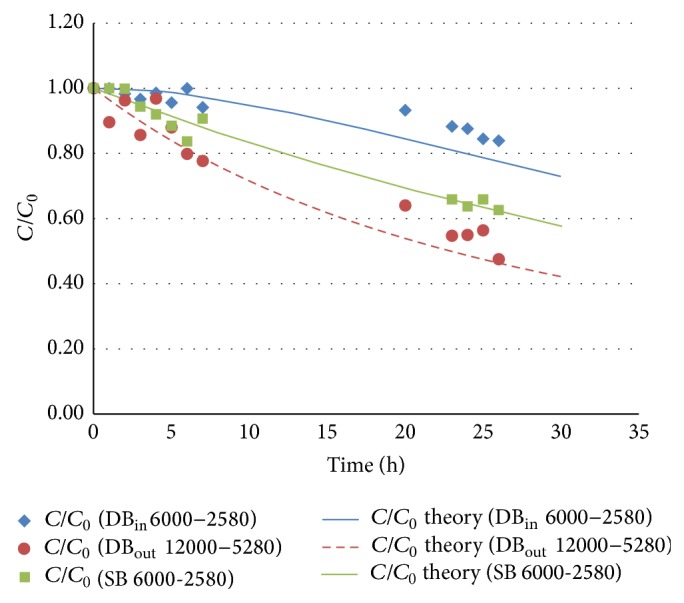
Concentration profile of NH_3_ over time for double bag: experimental data (dots) versus theoretical trend (continuous line). The inner Nalophan bags (DB_in_ 6000–2580) have *V*_*n*_ = 6000 cm^3^ and *S*_*n*−1_ = 2580 cm^2^, while the outer (DB_out_ 6000–5280) have *V*_*n*_ = 6000 cm^3^ and *S*_*n*_ = 5280 cm^2^.

As shown in Figure 3, the concentration trend expressed as *C*/*C*_0_ can be plotted against time for the double bag case. Experimental data show a good agreement with the expected trend in accordance with the system of (19). In order to compute the trend described in theory by the model's equations (19), the specific diffusivity parameter of NH_3_ was employed through Nalophan (*D*_NH_3__^Nalo^) as evaluated by Sironi et al. that is equal to 2.38∗10^−8^ cm^2^/s [24].

## 5. Conclusions

In the present work diffusive phenomena were investigated for a new sampling bag configuration that is the so-called double bag, capable of reducing the trans polymeric membrane concentration difference (Δ*C*) of the analyte. In the mathematical formulation of Fick's law, the (Δ*C*) is a parameter critical to the estimation of the diffusive material flux (*j*). It is possible to modify this (Δ*C*) utilizing two concentric sampling bags, both filled with the same gaseous mixture so to realize a cavity between the storage inner bag (DB_in_ 6000–2580) and the external environment capable of reducing the (Δ*C*).

The examination of the results highlights that this new configuration for the storage device is effective for the reduction of ammonia losses from the bag. Comparing the inner bag losses with those of the single bag, since the two are commensurate in terms of both tests conditions (*T*, *P*, *u*) and geometrical characteristics of the bag (*S*/*V*, *z*), it was observed that the inner bag of the double bag displays a 16% loss while the single bag a 37% loss. Thus, it can be concluded that acting on the (Δ*C*) it is possible to achieve a gross reduction of 57% in the ammonia leakage due to diffusion.

As a final remark, it is important to highlight once more that the structure of the double bag has with no doubts several advantages with respect to the common single bags as far as gaseous odorous mixtures storage is concerned. The double bag configuration is expected to reduce losses observed for other VOCs using the same equation, the same bags configuration, and specific diffusion coefficient.

## Figures and Tables

**Table 1:** Experimental data relevant to NH_3 _diffusion over time in Nalophan bags: the inner double bags (DB_in_ 6000–2580) have *V*_*n*_ = 6000 cm^3^ and *S*_*n*−1_ = 2580 cm^2^, the outer double bags (DB_out_ 6000–5280) have *V*_*n*_ = 6000 cm^3^ and *S*_*n*_ = 5280 cm^2^, and the single bag is with *V*_*n*_ = 6000 cm^3^ and S_n_ = 2580 cm^2^.

Time [h]	DB_in_ 6000–2580		DB_out_ 6000–5280		SB 6000–5280	
	ppm	NH_3 loss%_inner_	ppm	NH_3 loss%_outer _	ppm	NH_3 loss%_
0	55000	0,00	55000	0,00	54714	0,00
1	54973	0,05	49283	10,39	54698	0,03
2	54075	1,68	52952	3,72	54652	0,11
3	53176	3,32	47120	14,33	51625	5,65
4	54217	1,42	53264	3,16	50334	8,00
5	52569	4,42	48394	12,01	48393	11,55
6	54971	0,05	43929	20,13	45778	16,33
7	51775	5,86	42715	22,34	49613	9,32
⋮	⋮	⋮	⋮	⋮	⋮	⋮
20	51286	6,75	35201	36,00	⋮	⋮
⋮	⋮	⋮	⋮	⋮	⋮	⋮
23	48553	11,72	30089	45,29	36034	34,14
24	48180	12,40	30233	45,03	34878	36,25
25	46453	15,54	31007	43,62	36033	34,14
26	46137	16,11	26136	52,48	34248	37,41
